# Another in the Fold

**Published:** 1900-09

**Authors:** 


### ANOTHER IN THE FOLD.

VETERINARY journalism, with its charms and fascination, has budded forth in the newer Mecca of Association circles ot Michigan.

It comes forth full of fire and with a show of metal steeled for every fire, and an armor-plate that will win victory from every antagonist.

It is backed by one of the newer veterinary schools, and its battles will be theirs to fight. Its enemies theirs to conquer and humiliate in defeat.

It will fight the alien veterinarian of our land and cry aloud for the protection of the American citizen veterinarian.

It will urge legislation within the borders of its own State and in every other Commonwealth that shall drive out those who have ignored citizenship of these United States.

It will sustain and uphold State officials in their work of investigation only when their work will bear personal scrutiny and the test of its editors.

It is a good deal like a rooster who went forth "'lectioneering for a licking," and in these warm days of disputes it will be very apt to be accommodated.

It is modest only in its typographical make-up, but one so full of vigor for fighting its way is sure to attract attention and find enemies ready for battle on every side.

It may find the path of veterinary journalism strewn with ungarnered laurels, its victories and triumphs a crown of thorns, and it will surely realize the truth of that Ohio man's fate who trifled with the heels of a mule; he wasn't so all-fired good-looking thereafter, but he had a durned sight more good sense.

---

THE programme of papers and the clinics and other attractions of the Missouri State Veterinary Association meeting, October 3d and 4th, at St. Louis, promises to make it one of the most important meetings ever held in that State. State legislation will be considered and a legislative act will be considered to be presented at the next session of the Legislature. The JOURNAL wishes the profession a successful meeting, and urges all Missouri veterinarians to attend the meeting.

The Messrs. J. B. Lippincott Company, of Philadelphia, who have placed a number of books upon the market of special interest to the veterinarian, among others the *Exterior of the Horse* will erect a magnificent publishing-house on East Washington Square, tearing-down for this purpose several old mansions closely associated with the early history of the "City of Brotherly Love."

